# Genomic Landscape of Metastatic Lymph Nodes and Primary Tumors in Non-Small-Cell Lung Cancer

**DOI:** 10.3389/pore.2022.1610020

**Published:** 2022-06-16

**Authors:** Bing Chen, Rutao Li, Junling Zhang, Lin Xu, Feng Jiang

**Affiliations:** ^1^ The Affiliated Cancer Hospital of Nanjing Medical University, Nanjing, China; ^2^ Department of Thoracic Surgery, Jiangsu Cancer Hospital, Nanjing, China; ^3^ The Medical Department, 3D Medicines Inc., Shanghai, China

**Keywords:** gene mutation, ARID1A, non-small-cell lung cancer, metastatic, CTNNB1

## Abstract

**Objective:** To investigate the genetic mutation characteristics of non-small cell lung cancers (NSCLC) with and without lymph node metastasis.

**Methods:** Primary lesions and metastatic lymph node lesions of 36 Chinese NSCLC patients were tested for somatic mutations, tumor mutation burden, phylogenetic and clonal evolutional analysis using a 1021-gene panel by next-generation sequencing (NGS) with an average sequencing depth of 671X.

**Results:** In this study, eighteen patients with lung adenocarcinoma (LUAD) and 18 with lung squamous cell carcinoma (LUSC) were included. Different groups had distinct characteristics of gene mutations. CTNNB1 gene mutations were only present in Nome_LC LUAD patients (*p* < 0.05). ARID1A mutation was however the only gene with significant alterations (*p* < 0.05) in Nome_LC in LUSC. Phylogenetic trees of mutated genes were also constructed. Linear and parallel evolutions of metastatic lymph nodes were observed both in LUAD and LUSC.

**Conclusion:** LUSC exhibited more genetic mutations than LUAD. Intriguingly, there was significant difference in gene mutations between Meta_LC and Nome_LC. CTNNB1 gene alteration was the key mutation in LUAD that seems to promote proliferation of the tumor and then determine T stage. On the other hand, proliferation of the tumor was characterized by ARID1A missense mutation in LUSC, thus influencing the T stage as well. Lymph node metastasis could display both linear and parallel evolutionary characteristics in NSCLC. Different metastatic lymph nodes might have exactly the same or different mutated genes, underlining the heterogeneous genomic characteristics of these cancer types.

## Introduction

Lung cancer is the most prevalent and leading cause of cancer related deaths, particularly in developing countries such as China [1]. About 85% of new lung tumors are non-small cell lung cancers (NSCLC) [1]. In addition, nearly half of NSCLCs are metastatic types at the time of diagnosis, whose overall 5-year survival rate is only 5.5% [2]. This underscores the need to unravel events underlying tumorigenesis of NSCLC. The most common form of NSCLC metastasis is lymph node metastasis [3]. Research shows that Lymph node metastasis is the most important factor affecting the prognosis of primary lung cancer [4]. The introduction of low-dose CT has enhanced early diagnosis of early stage lung cancer. Interestingly, pathological analyses have identified lymph node metastasis in many patients with small primary tumors. Conversely, some other patients with large primary tumors do not exhibit lymph node metastases. NSCLC with lymph node metastasis is associated with poorer prognosis, relative to NSCLC not displaying this pathology [5]. Our previous research revealed that approximately 14.6%–16.5% of patients with stage T1 NSCLC exhibit lymph node metastasis [6, 7]. Although researches have elucidated the cellular and molecular mechanisms involved in NSCLC development, the genetic divergence and phylogenetic structure of lymph node metastases in small primary tumors remain largely unknown.

Next-generation sequencing (NGS) of tumor biopsies has greatly expanded our knowledge on somatic mutations in NSCLC such as the deep genetic heterogeneity in tumors beyond epidermal growth factor receptor (EGFR) changes [8]. Genomic classification may improve clinical management and corresponding prognosis. However, the clinical value of genomic changes relies on understanding the interaction between the genetic alterations, treatment received, heterogeneity and the dynamics of mutations in disease evolution. This underscores the need to describe the genetic characteristics of NSCLC with and without lymph node metastasis, not only to understand the lesional changes, but with a view of developing personalized treatment.

Herein, we report sequence analyses of tumor tissues from 37 NSCLC patients. For the first time, we analyzed the genetic variation pattern and clonal evolution pattern of NSCLC with and without lymph node metastasis. Identification of metastatic factors to select patients with a poor prognosis and development of tailored treatment strategies are then advisable.

## Methods

### Study Design

Between January 2013 to December 2016, 18 non-metastatic NSCLC (Nome_LC) patients with tumor size over 3 cm, and 18 T1 NSCLC patients with lymph node metastases (Meta_LC) from Jiangsu Cancer Hospital were enrolled for this study. A total of 59 NSCLC tissues including primary lesion and metastatic lymph nodes were extracted from the 36 patients, fixed in formalin and embedded in paraffin (FFPE) for somatic mutations analyses. The pathological FFPE findings were validated using hematoxylin and eosin (HE) staining. The age, gender, TNM stage, tumor histology and smoking history of the patient were all recorded.

### Targeted Next-Generation Sequencing

An NGS approach was performed on genomic DNA isolated from FFPE surgically resected NSCLC tissue samples. Tissues smaller than 1 mm or with less than 20% tumor cells were all excluded. A targeted pan‐cancer panel with 1021 cancer related genes was conducted and was performed on the Hiseq NGS platforms (Illumina Inc., San Diego, CA, United States) using of base substitutions, indel, copy number variations (CNV) and DNA arrangement analysis. Targeted capture sequencing revealed a median effective depth of coverage of 671× in the tissue samples. The detailed genes for the 1021 cancer-gene panel were provided in Supplementary Table S1.

### Tumor Mutational Burden

The tumor mutation burden (TMB) of a tumor a tumor was calculated based on the number of non-synonymous somatic mutations (single nucleotide variants and small insertions/deletions) per mega-base in coding regions. All germline variants were filtered using paired blood controls.

### Phylogenetic Analysis

Phylogenetic analysis of tumor genes sequences was analyzed using the ape package. For each patient, an optimal phylogenetic tree was inferred using the maximum parsimony method. Candidate driver mutations were mapped to the trunk and branches of each phylogenetic tree to illustrate the molecular processes underlying NSCLC metastasis.

### Clonal Evolution Analysis

Subclones generated from SNVs were clustered sing PyClone. The optimal tree was generated using the clustering outputs. The analysis was performed using iterative version of citup. The fish plot and phylogenetic tree of each patient were constructed using timescape.

### Statistical Analyses

Categorical variables were expressed using number and proportions. Somatic mutation data of NSCLC patients from TCGA database were downloaded from cBioPortal [8]. The relationships between groups were analyzed using Pearson’s chi-square or Fisher’s exact test, based on the Yates continuity correction coefficient, with *p* < 0.05 considered statistically significant. Data was analyzed using SPSS22.0 software (SPSS, Inc., Chicago, IL, United States).

## Results

### Patients and Clinical Characteristics

The baseline characteristics of the 36 NSCLC patients included in this study are summarized in Table 1. Overall, 59 NSCLC tissues were extracted and analyzed. Of the 36, 27 patients (75%) were male, with majority (83.33%) above 65 years old. The patients were divided into two groups based on the absence or presence of lymph node metastasis. We found 18 patients with one or two lymph nodes metastases (Meta_LC group). The median tumor size in these patients was 2.5 cm (range 1.0–3.0 cm). The rest 18 patients (50%), had no lymph node metastasis (Nome_LC group). Nine tissues were selected from each group of lung adenocarcinoma (LUAD) and lung squamous cell carcinoma (LUSC) for further analyses. The combined median tumor size of Meta_LC group was 2.5 cm (range 1.0–3.0), almost three times the size of the smallest tumor in the Nome_LC group (7.1 cm, range from 5.0 to 12.0). There was no distant metastasis in any of the cases (M stage: M0).

**TABLE 1:** Patient baseline characteristics.

Lung Cancer				
Variable		Meta_LC	Nome_LC	Total
All patients		18 (100%)	18 (100%)	36 (100%)
Age				
	≤65 years	3 (16.67%)	3 (16.67%)	6 (16.67)
	≥65 years	15 (83.33%)	15 (83.33%)	30 (83.33%)
Gender				
	Female	4 (22.22%)	5 (27.78%)	9 (25.00%)
	Male	14 (77.78%)	13 (72.22%)	27 (75.00%)
Smoking				
	Yes	6 (33.33%)	8 (44.44%)	14 (13.89%)
	No	12 (66.67%)	10 (55.56%)	22 (61.11%)
Tumor histology				
	AD	9 (50.00%)	9 (50.00%)	18 (50.00%)
	SCC	9 (50.00%)	9 (50.00%)	18 (50.00%)
T stage				
	T1	18 (100%)	0	18 (50.00%)
	T2	0	2 (11.11%)	2 (5.55%)
	T3	0	10 (55.56%)	10 (27.78%)
	T4	0	6 (33.33%)	6 (16.67%)
N stage				
	N0	0	18 (100%)	18 (50.00%)
	N1	8 (44.44%)	0	8 (22.22%)
	N2	10 (55.56%)	0	10 (27.78%)
M stage				
	M0	18 (100%)	18 (100%)	36 (100%)
	M1	0	0	0
Tumor size (cm)				
	Range	[1.0, 3.0]	[5.0, 12.0]	
	Median	2.5	7.1	

AD, adenocarcinoma; SCC, squamous cell carcinoma; cm, centimetre.

### The Genetic Characteristics of Nome_LC, Meta_LC and LN Groups

**FIGURE 1:**
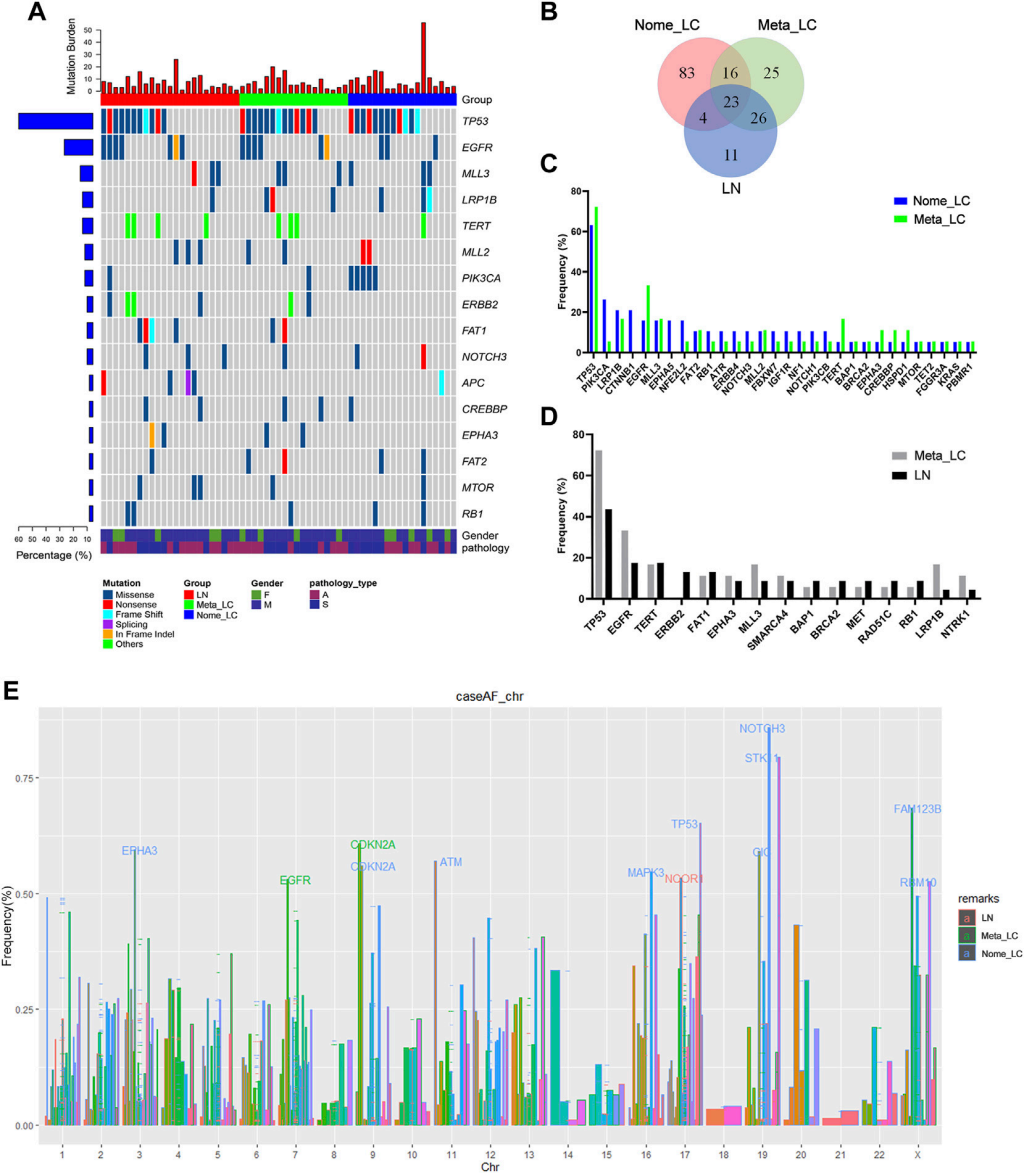
The genomic landscape and molecular characteristics. **(A)** The genomic landscape of tissue sample. **(B)** The Venn diagram of three groups, shows the number of mutated genes. **(C)** The frequency mutated gene in Nome_LC and Meta_LC group. **(D)** The frequency mutated gene in Meta_LC and LN group. **(E)** Mutated genes in three subgroups among different chromosomes, genes with high level frequency are highlighted.

Next-generation sequencing of the 59 samples uncovered a pan-cancer panel of 1021 tumorigenesis related genes. The average sequencing depth of DNA extracted from the aforementioned tissues was 671×[9]. The somatic mutations investigated included small nucleotide variants (SNVs), copy number variants (CNVs), short insertions or deletions (indels) and structure variations (SVs). Overall, we identified 486 somatic mutations, with the majority in TP53 (58.3%), EGFR (21.7%), MLL3 (13.3%), LRP1B (13.3%) and TERT (13.3%) genes (Figure 1A). We downloaded sequence data for 68 LUAD and 70 LUSC patients from The Cancer Genome Atlas (TCGA) for further analyses (Supplementary Figure S1A). The top five most common alterations were not quite the same as genes identified in our study. For Nome_LC group, mutations were detected in 126 genes. Comparatively, there were 51 gene variations in the Meta_LC group, 26 genes of which were also detected in the LN group. Notably, mutations in 11 genes were unique to the LN group alone (Figure 1B). Mutation frequency of common driver genes was quite different between Nome_LC and Meta_LC groups, although it was not statistically significant (Figure 1C).

Intriguingly, there was a clear distinction in the frequency and type of mutant genes between Nome_LC and Meta_LC groups. For instance, for Nome_LC group, the majority of mutations occurred in the TP53 (66.7%), PIK3CA (27.8%), LRP1B (22.2%), CTNNB1 (22.2%), and EGFR (16.7%) genes. However, TP53 (72.2%), EGFR (33.3%), LRP1B (16.7%), MLL3 (16.7%) and TERT (16.7%) were the top most mutated genes in the Meta_LC group (Figure 1D). EGFR (33.3% vs. 15.8%) and TERT (16.7% vs. 5.2%) were the most mutated genes in the Meta_LC group (Supplementary Table S2). In LN group, *TP53* (43.5%), *EGFR* (17.4%), *TERT* (17.4%), *ERBB2* (13.0%) and *FAT1* (13.0%) were the top most mutated genes (Figure 1D). Notable, the difference in the most common mutations between Meta_LC and LN group was very small (Supplementary Table S3).

We further compared the distribution of the most frequently altered genes in Meta_LC, Nome_LC and LN groups. The highest gene mutations were observed in the Meta-ca group. Interestingly, there was a significant difference in the distribution of altered genes between Nome_LC and LN group (Supplementary Figure S1C).

On each chromosome, the gene was marked when its mutation frequency was greater than 50% and we defined it as having a high mutation frequency. With regard to high-level frequency of mutated genes among the three subgroups, Meta_LC group exhibited two high-level frequency mutations (*EGFR*, *CDKN2A*), whereas in LN group, high-level frequency gene mutation was only observed in one gene (*NCOR1*). However, high-level frequency mutations were observed in ten genes (*EPHA3*, *CDKN2A*, *ATM*, *MAPK3*, *TP53*, *NOTCH3*, *STK11*, *CIC*, *FAM123B*, *RBM10*) in the Nome_LC group (Figure 1E). Overall, patients with large primary tumor size and those without lymphatic metastasis displayed more high-level gene mutations. Even so, precise mechanism underlying the phenomenon needs further investigation.

Further analyses reveled that patients with higher Tumor Mutation Burden (TMB/Tumor Mutation Load (TML)) responded better to tumor immunotherapy [10]. Compared to patients in Meta_LC and LN groups, those in Nome_LC group presented with higher mutation burden. However, no significance difference in median TMB was seen between primary and metastatic tumors (Supplementary Figure S1B).

### Genetic Variation in LUAD and LUSC Tissues

Sequence data for 562 LUAD and 470 LUSC patients (1032) was downloaded from the TCGA database. Additional validation data for 5597 patients was downloaded from the Memorial Sloan Kettering Cancer Center (MSK) database. As shown in Supplementary Figure S2, about 90% of high frequency mutated genes both in LUAD (Supplementary Figure S2A) and LUSC (Supplementary Figure S2B) detected in this study were indentified in cancer tissues of patients in the TCGA and MSK database, which means the results obtained by panel sequencing were reliable.

**FIGURE 2:**
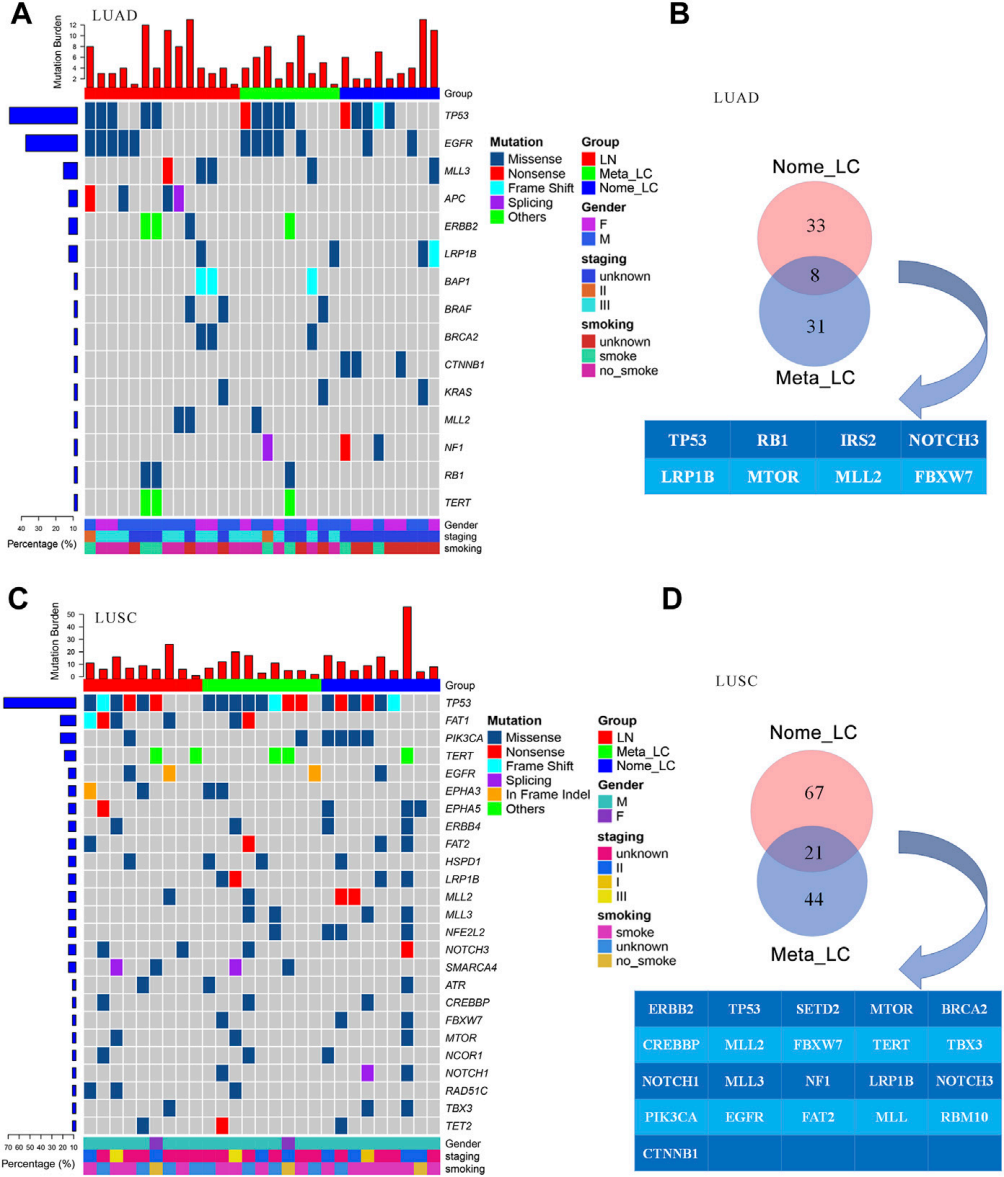
The genomic landscape of LUAD and LUSC. **(A)** Top 15 significantly mutated genes in LUAD, samples were ordered based on their somatic non-synonymous mutation burden (top panel) and genes were ranked by mutation frequencies (left panel). Gender and age are annotated in the bottom panel. **(B)** The Venn diagram of two groups in LUAD, shared genes are presented blow. **(C)** Top 25 significantly mutated genes in LUSC. **(D)** The Venn diagram of two groups in LUSC, shared genes are presented blow.

Further comparisons in genetic aberrations between LUAD and LUSC of our data were made. We identified 173 somatic mutations in 32 lung adenocarcinoma tissues (Figure 2A). The top most altered genes included *TP53*, *EGFR*, *MLL3*, *APC*, *ERBB2*, *LRP1B*, *BAP1*, *BRAF*, *BRCA2* and *CTNNB1*. Nome_LC and Meta_LC groups only shared eight mutated genes (Figure 2B). In addition, there was no significance difference in mutation frequency between the two groups (Supplementary Table S4). In 27 lung squamous carcinoma tissues, we identified 302 somatic mutations (Figure 2C). The top five most commonly altered genes included *TP53*, *FAT1*, *PIK3CA*, *TERT* and *EGFR* (Supplementary Table S5). Meta_LC and Nome_LC groups shared 21 mutated genes, conflicting findings of the TCGA analyses (Supplementary Figures S2C,D). Interestingly, the rate of mutations in squamous cell carcinoma tissues was significantly high, relative to adenocarcinoma tissues. The average number of mutations per LUSC and LUAD tissue was 8.1 and 5.4, respectively (Figures 2A,C). These findings demonstrate the marked mutational distinction between LUSC and LUAD tissues. However though, LUSC exhibits greater gene mutations.

### Genes Related to T Stage in LUAD and LUSC

Given that small primary tumor (T1) may present with lymph node metastasis, and relatively large primary tumors (>T2) can present without lymph node metastasis, we sought to identify the genetic mutations associated with lymph node metastasis or proliferation of primary tumor cells.

**FIGURE 3:**
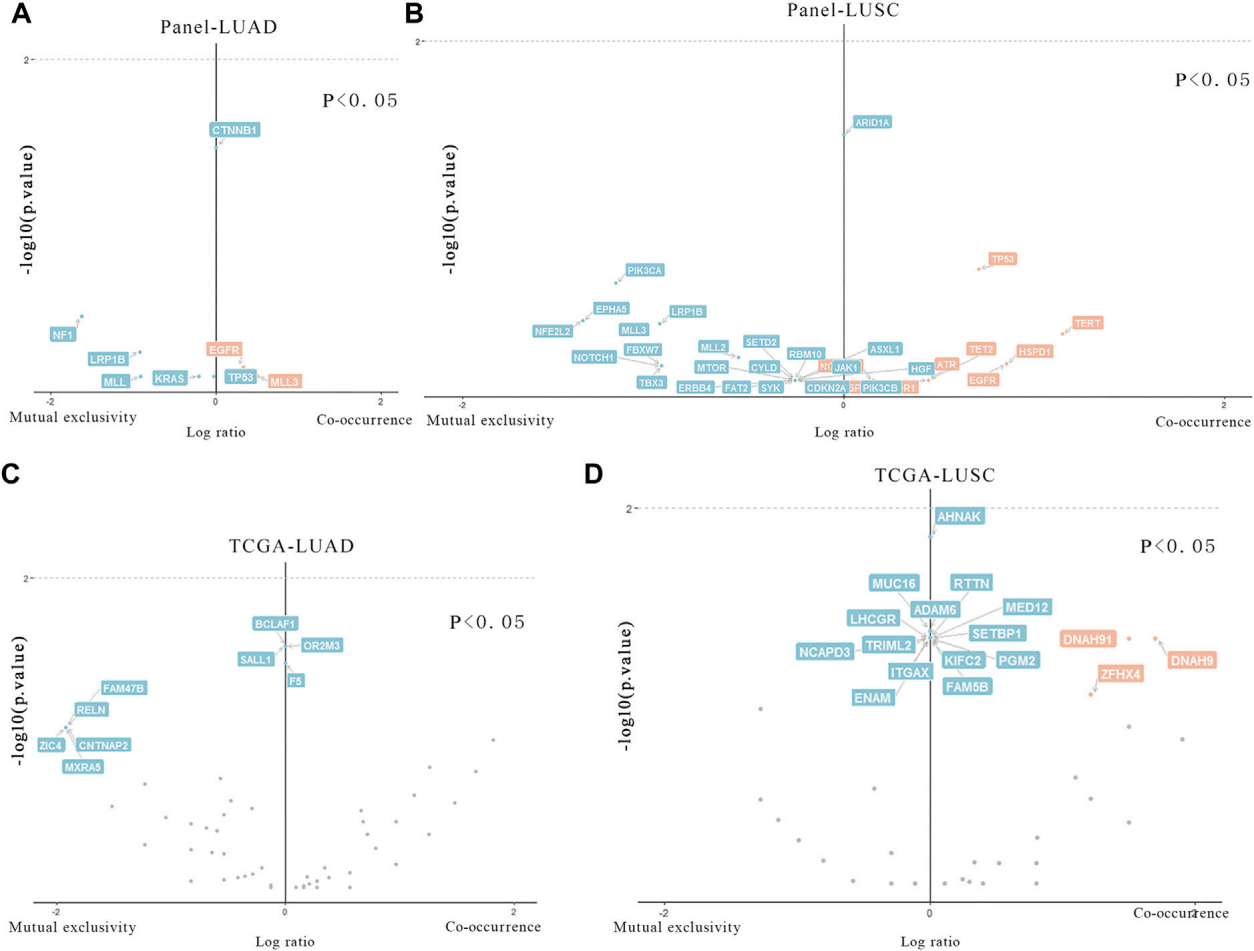
Particular genes related to T stage in LUAD and LUSC. **(A,B)** Genes in our data that are statistically different in two groups of LUAD and LUSC. **(C,D)**. Genes in TCGA data that are statistically different in two groups of LUAD and LUSC.

The differences of mutated genes were analyzed in Meta_LC and Nome_LC group. We found that mutations in *CTNNB1* was significantly (*p* < 0.05) different and only presented in Nome_LC group in LUAD (Figure 3A). Further analysis of 27 LUSC tissues only identified mutations in *ARID1A* genes (Figure 3B) among patients in the Nome_LC group. Analysis of data downloaded in the TCGA database identified significant mutations in *KCNH7*, *UNC9*, *ADAMTLS3*, *HCN1*, *AHNAK2*, *EPHA7*, *MXRA5*, *CTNNA2* and *SEMA5A* (*p* < 0.05) in the two groups of LUAD (Figure 3C). and 17 genes (*E4F1*, *EPYC*, *FGF12*, etc.) were detected in TCGA data (Figure 3D). In general, findings of TCGA data analyses were substantially different from those generated from analysis of our data. This discrepancy may have arisen from numerous sources.

### Phylogenetic Trees and Mutation Clone Landscape of Lymph Node Metastasis

**FIGURE 4:**
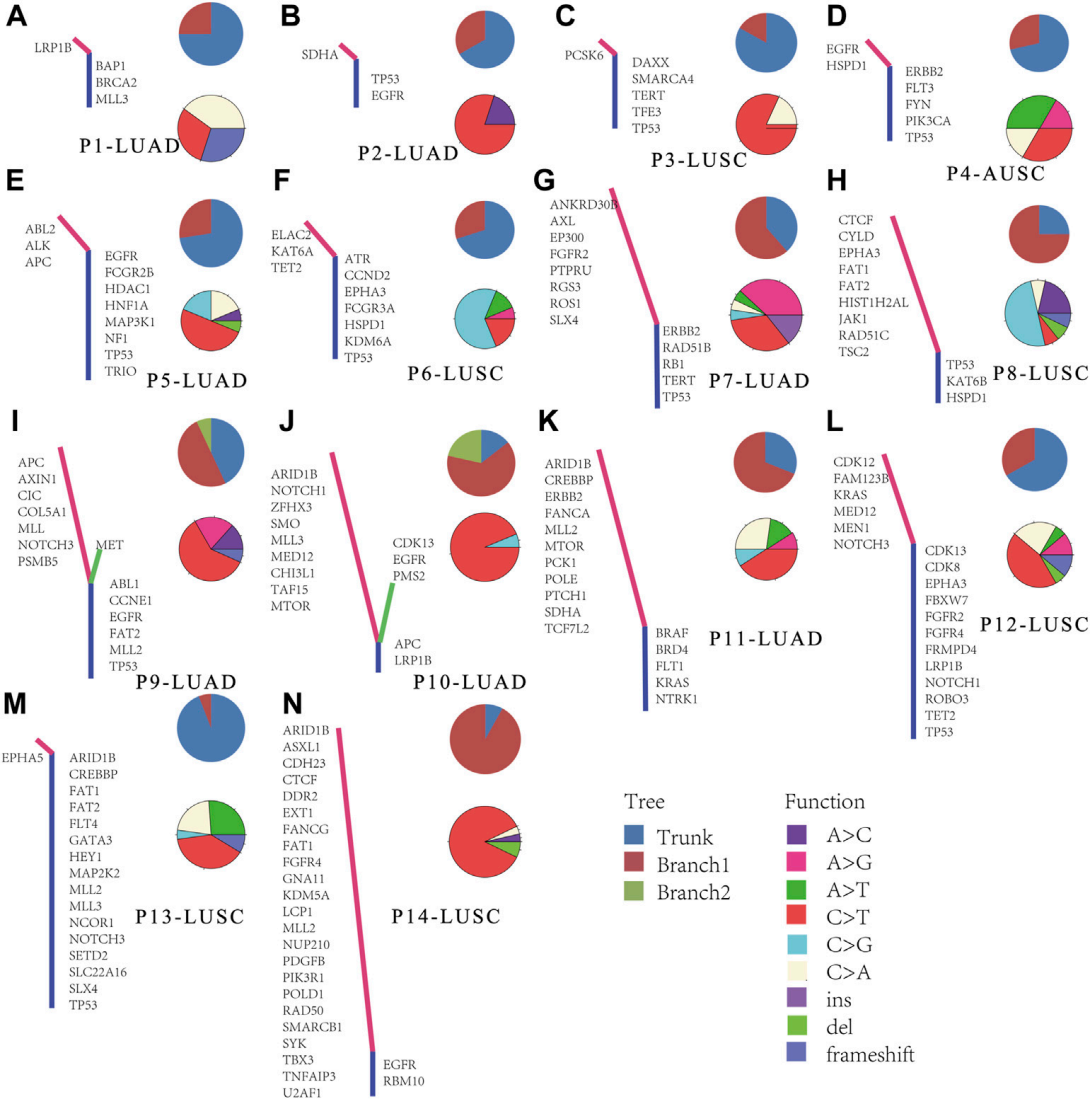
Phylogenetic analysis in paired primary tumors and metastatic lymph nodes. **(A–N)** Phylogenetic trees of cases, trunk means the genes mutated both in primary tumor and all detected lymph nodes, each branch means the mutated genes in every lymph node sample.

To further understand the evolution of lymph node metastasis, we compared genetic aberrations in primary tumors and metastatic lymph node in the Meta_LC group. The trunks of phylogenetic trees represented genes with mutations both in primary tumors and all lymph nodes. Branches represent mutated genes with lymph nodes only (Figure 4). In some cases, we observed high similarity in genetic variation between metastatic lymph nodes (Figure 4M) and their paired primary tumors. This suggest that metastatic lymph nodes “drifted” from the primary tumor relatively late in the evolutionary process. However, other genetic alterations were highly distinct between groups (Figure 4N), demonstrating early “drift” between evolutionary groups. Overall, we identified two distinct evolutionary models both in LUAD and LUSC. These results suggest that metastatic lymph nodes followed the linear progression model and parallel progression model. In the cases of the primary tumor with two metastatic lymph nodes, there were also different evolutionary patterns (Figures 4A,G,I–K). In one pattern, two metastatic lymph nodes shared exactly the same branch (Figure 4A). In another pattern, two metastatic lymph nodes shared a trunk but have distinct branches (Figures 4I,J). And in the other pattern, similarity was observed between one metastatic lymph node and its paired primary tumor, the other metastatic lymph node however, was quite different between them (Figures 4G,K).

**FIGURE 5:**
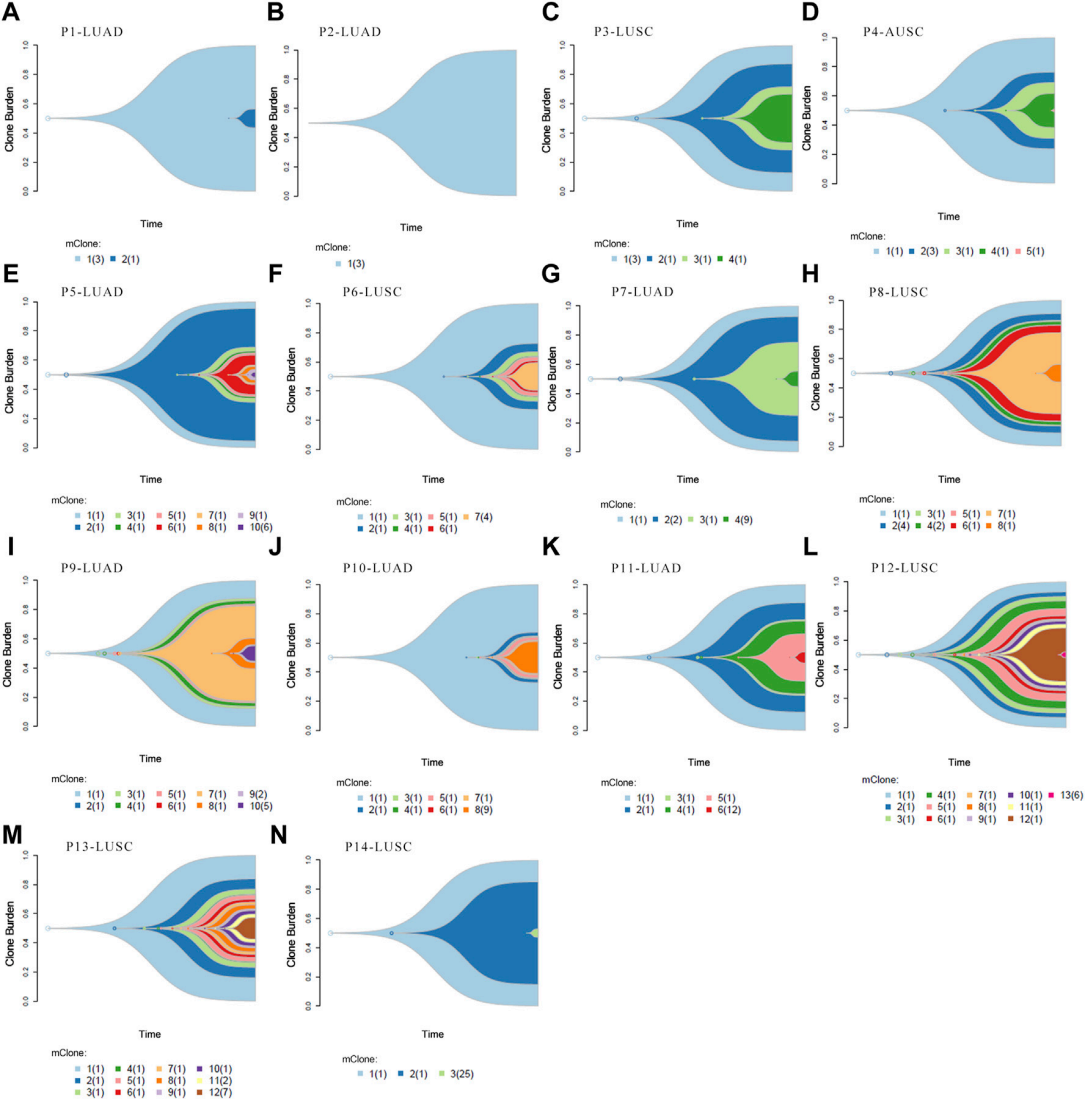
Clonal evolution analysis. **(A–N)** Fish plots constructed by timescape using pyclone algorithm. The relative time of genes’ occurrence are inferred from different clonal loads. Colors indicate different clones.

Clonal analysis of altered genes in 14 NSCLC lymphatic metastases revealed the hypothetical subclonal development process and cancer evolution. The mutant gene with the maximum load was regarded as having a cell proportion of 1 (100%), and the other genes decreased in turn. The mutation load was negatively proportional to the occurrence time. The evolution patterns of subclones differed from patient to patient, characteristic of intratumor heterogeneity (Figure 5). It’s worth noting that several LUAD tissues displayed fewer or slightly more mutated (Figures 5A,B,E,I). This phenomenon corresponded with low level TMB. However, there were greater mutations in most LUSC, which corresponded with high TMB (Figures 5H,L,M).

## Discussion

Herein, we assessed the genetic alterations in NSCLC patients with and without lymph node metastasis, with the aim of unraveling the characteristics of driver gene mutations in lymph node metastasis.

Metastasis results from dynamic interactions between tumor cells and new microenvironment [11]. Metastasis was thought to be advanced primary tumors. Although this might be true for some tumors, recent evidence suggested that progression of primary and metastasis can occur concurrently. Simply put, metastasis could occur in primary/newly diagnosed early cancers [12]. However, whether genetic blueprints of metastatic lymph nodes differ from those of primary tumors had remained to be validated.

Sequence analysis identified highest mutation frequencies in *TP53*, *EGFR*, *MLL3*, *LRP1B* and *TERT* genes in of NSCLC tissues. Further analysis of TCGA data revealed that TP53 was the only shared gene with high frequency mutations in NSCLC tissues. This might be attributed to ethnical genetic differences and the small amount of our data. The number of mutated genes in Nome_LC group, Meta_LC group and LN group were ranked from highest to lowest in order. This might have resulted from equilibrium checks between oncogenes and tumor suppressor genes, which to a certain extent, modulate progression and tumor metastasis of malignant tumors. However, there were very fewer mutated genes in metastatic lymph nodes, which may only be related to metastasis of primary lesions.

The mutant genes in metastatic lymph nodes were comparable to those of corresponding primary tumor, demonstrating the clonal evolution of the tumor. At the same time, we found marked difference in mutated genes between LN and the Nome_LC cancers. Most genes either appeared in the Nome_LC or LN group alone, but rarely both. These finds suggest certain unique gene mutations promote proliferation and metastasis of specific tumors, which influence the T and N stages.

The frequencies of genetic mutations in LUAD and LUSC were consistent with those in identified in independent cohorts in the TCGA and MSK databases [13]. By comparing our data with those of TCGA and MSK, it was confirmed that the pan-cancer panel we selected with 1021 genes could cover common mutated genes. This proved that the panel we used to detect somatic mutations was credible. However, Genes with Our results high-frequency mutations varied between LUAD and LSCS tissues. Contrarily, analysis of TCGA data reveled substantial similarity in the top 5 high frequency mutant genes between LUAD and LSCS tissues. For LUSC, the majority of the high mutant genes were similar both in Meta_LC and Nome_LC group. In LUAD however, the two groups share less than one quarter of the mutated genes. Contrarily, based on TCGA data, there was very little difference in top five most common mutated genes between LUAD and LUSC. This disparity may have emanated from the quality of sequence transcripts. Better sequencing results could be obtained by using fresh tissues. The differences between our own and TCGA data were probably due to non-homogeneous sample population and racial differences. Meanwhile, sequencing platforms with different coverages and depths may introduce bias when calling large structural variants In addition, our findings were consistent with previous studies, in which there were more mutant genes in lung squamous cell carcinoma tissues, relative to lung adenocarcinoma [14].

Analysis results revealed that mutations in *CTNNB1* were only appeared in Nome_LC but not Meta_LC group of LUAD. We thought that missense mutation of this gene might mediate proliferation of tumors, thus influencing the T stage. *CTNNB1* is a key regulator of the Wnt signaling pathway. beta-catenin 1 interacts with E-cadherin and actin cytoskeleton to mediate cell-cell adhesion [15]. Research shows that over-expression of *CTNNB1* promotes metastasis of colorectal cancer [16] and colon cancer [17]. Mutations in *CTNNB1* gene over-activate the Wnt signaling pathway in human adrenocortical tumors [18, 19]. To some extent, mutations in the *CTNNB1* gene increases the risk of malignant transformation [18]. *ARID1A* is another gene differently altered between Meta_LC and Nome_LC in LUSC. Research shows that *ARID1A* suppresses development and progression of tumors [20]. Interestingly, wild-type *ARID1A* gene has been reported in an individual with endometrial cancer tissues. However, the deleterious *ARID1A* gene mutate was found in a metastatic lesion [21]. Loss of *ARID1A* is related to size, infiltration depth, lymph node metastasis and poor prognosis of gastric cancer [22]. This actually supportted our suspect that missense mutation of *ARID1A* might mediate proliferation in LUSC, thus influencing the T stage as well. Somewhat disappointingly, we did not see characteristic gene mutations in LUAD Meta_LC group nor LUSC Meta_LC group. For patients with *CTNNB1* mutation in LUAD and *ARID1A* mutation in LUSC, it may be more important to focus on local recurrence.

We then constructed an evolutionary tree using data of 14 patients with lymph node metastases (7 LUAD and 7 LUSC). Previous studies showed that lung squamous cell carcinoma displays more mutated genes. Evolution of tumor occurred through numerous models. For instance, brain metastases more often displayed parallel evolution, whereas liver metastases mostly undergo linear evolution [23]. In this study, we found lymph node metastases display both linear and parallel evolutionary patterns. On the other hand, different metastatic lymph nodes from the same patient could have exactly the same or different mutated genes. These findings demonstrated the need for assessing lymph node metastases in cancer patients.

Our findings notwithstanding, more data is needed to uncover the NSCLC genotype associated with lymph node metastases. However, findings of this study lay the necessary foundation for deeper and extensive exploration of lymph node metastases and corresponding genotypes. Regarding limitations, first, the sample size used in this study was relatively small, which casts doubts on the credibility of our findings. Studies utilizing larger data are therefore necessary to validate findings of this study. Second, we lacked prognostic data though we had tried our best to return to patients’ prognostic information. In general, we highlighted the genetic signature of T1 NSCLC patients with lymph node metastasis and T2-4 NSCLC patients without lymph node metastasis. There are more genetic mutations in LUSC. In LUAD however, the differences in mutations between the Meta_LC and Nome_LC groups are greater. *CTNNB1* mutation and *ARID1A* mutation promoted proliferation of LUAD and LUSC, respectively, thus affecting the T stage. Lymph node metastasis exhibits both linear and parallel evolution. Mutated genes in metastatic lymph nodes are not constant. They may be exactly the same or very different between different sub-types.

## Data Availability

The original presentations in the study are included in the article/Supplementary Material, further inquiries can be directed to the corresponding authors. The raw data supporting the conclusion of this article will be made available by the authors, without undue reservation.

## References

[1] Siegel RL Miller KD Jemal A. Cancer Statistics, 2020. CA Cancer J Clin (2020) 70:7–30. 10.3322/caac.21590 31912902

[2] Howlader N Noone AM Krapcho M Miller D Bishop K Kosary CL SEER Cancer Statistics Review, 1975-2014. Bethesda, MD: National Cancer Institute (2017). Based on November 2016 SEER Data Submission, posted to the SEER web site.

[3] Xu S Yang J Xu S Zhu Y Zhang C Liu L Lymphatic Vessel Density as a Prognostic Indicator in Asian NSCLC Patients: A Meta-Analysis. BMC Pulm Med (2018) 18:128. 10.1186/s12890-018-0702-9 30081883 PMC6091207

[4] Adachi Y Nakamura H Kitamura Y Taniguchi Y Araki K Shomori K Lymphatic Vessel Density in Pulmonary Adenocarcinoma Immunohistochemically Evaluated with Anti-podoplanin or Anti-D2-40 Antibody is Correlated with Lymphatic Invasion or Lymph Node Metastases. Pathol Int (2007) 57:171–7. 10.1111/j.1440-1827.2007.02077.x 17316411

[5] Sobin LH Compton CC TNM Seventh Edition: What's New, What's Changed. Cancer (2010) 116:5336–9. 10.1002/cncr.25537 20665503

[6] Chen B Wang X Yu X Xia Wj. Zhao H Li Xf. Lymph Node Metastasis in Chinese Patients with Clinical T1 Non‐small Cell Lung Cancer: A Multicenter Real‐world Observational Study. Thorac Cancer (2019) 10:533–42. 10.1111/1759-7714.12970 30666800 PMC6397906

[7] Cho S Song IH Yang HC Kim K Jheon S. Predictive Factors for Node Metastasis in Patients with Clinical Stage I Non-small Cell Lung Cancer. Ann Thorac Surg (2013) 96:239–45. 10.1016/j.athoracsur.2013.03.050 23673071

[8] Cerami E Gao J Dogrusoz U Gross BE Sumer SO Aksoy BA The cBio Cancer Genomics Portal: An Open Platform for Exploring Multidimensional Cancer Genomics Data. Cancer Discov (2012) 2:401–4. 10.1158/2159-8290.cd-12-0095 22588877 PMC3956037

[9] Zhang Y Chang L Yang Y Fang W Guan Y Wu A Intratumor Heterogeneity Comparison Among Different Subtypes of Non-small-cell Lung Cancer through Multi-Region Tissue and Matched ctDNA Sequencing. Mol Cancer (2019) 18:7. 10.1186/s12943-019-0939-9 30626401 PMC6325778

[10] Rizvi NA Hellmann MD Snyder A Kvistborg P Makarov V Havel JJ Mutational Landscape Determines Sensitivity to PD-1 Blockade in Non-small Cell Lung Cancer. Science (2015) 348:124–8. 10.1126/science.aaa1348 25765070 PMC4993154

[11] Hoshide R Jandial R. The Role of the Neural Niche in Brain Metastasis. Clin Exp Metastasis (2017) 34:369–76. 10.1007/s10585-017-9857-7 28905151

[12] Comen E Norton L Massagué J. Clinical Implications of Cancer Self-Seeding. Nat Rev Clin Oncol (2011) 8:369–77. 10.1038/nrclinonc.2011.64 21522121

[13] Zehir A Benayed R Shah RH Syed A Middha S Kim HR Mutational Landscape of Metastatic Cancer Revealed from Prospective Clinical Sequencing of 10,000 Patients. Nat Med (2017) 23:703–13. 10.1038/nm.4333 28481359 PMC5461196

[14] Jamal-Hanjani M Wilson GA McGranahan N Birkbak NJ Watkins TBK Veeriah S Tracking the Evolution of Non-small-cell Lung Cancer. N Engl J Med (2017) 376:2109–21. 10.1056/nejmoa1616288 28445112

[15] Willert K Jones KA. Wnt Signaling: Is the Party in the Nucleus? Genes Dev (2006) 20:1394–404. 10.1101/gad.1424006 16751178

[16] Tenbaum SP Ordóñez-Morán P Puig I Chicote I Arqués O Landolfi S β-Catenin Confers Resistance to PI3K and AKT Inhibitors and Subverts FOXO3a to Promote Metastasis in colon Cancer. Nat Med (2012) 18:892–901. 10.1038/nm.2772 22610277

[17] Wen J Min X Shen M Hua Q Han Y Zhao L ACLY Facilitates colon Cancer Cell Metastasis by CTNNB1. J Exp Clin Cancer Res (2019) 38:401. 10.1186/s13046-019-1391-9 31511060 PMC6740040

[18] Tissier F Cavard C Groussin L Perlemoine K Fumey G Hagneré A-M Mutations of β-Catenin in Adrenocortical Tumors: Activation of the Wnt Signaling Pathway is a Frequent Event in Both Benign and Malignant Adrenocortical Tumors. Cancer Res (2005) 65:7622–7. 10.1158/0008-5472.can-05-0593 16140927

[19] Durand J Lampron A Mazzuco TL Chapman A Bourdeau I. Characterization of Differential Gene Expression in Adrenocortical Tumors Harboring β-Catenin (CTNNB1) Mutations. J Clin Endocrinol Metab (2011) 96:E1206–E1211. 10.1210/jc.2010-2143 21565795

[20] Umphlett M Shea S Tome-Garcia J Zhang Y Hormigo A Fowkes M Widely Metastatic Glioblastoma with BRCA1 and ARID1A Mutations: A Case Report. BMC cancer (2020) 20:47. 10.1186/s12885-020-6540-1 31959133 PMC6971940

[21] Gibson WJ Hoivik EA Halle MK Taylor-Weiner A Cherniack AD Berg A The Genomic Landscape and Evolution of Endometrial Carcinoma Progression and Abdominopelvic Metastasis. Nat Genet (2016) 48:848–55. 10.1038/ng.3602 27348297 PMC4963271

[22] Zhu YP Sheng LL Wu J Yang M Cheng XF Wu NN Loss of ARID1A Expression is Associated with Poor Prognosis in Patients with Gastric Cancer. Hum Pathol (2018) 78:28–35. 10.1016/j.humpath.2018.04.003 29689245

[23] Jiang T Fang Z Tang S Cheng R Li Y Ren S Mutational Landscape and Evolutionary Pattern of Liver and Brain Metastasis in Lung Adenocarcinoma. J Thorac Oncol (2021) 16:237–49. 10.1016/j.jtho.2020.10.128 33188911

